# Effect of Common Pavements on Interjoint Coordination of Walking with and without Robotic Exoskeleton

**DOI:** 10.1155/2019/5823908

**Published:** 2019-10-01

**Authors:** Jinlei Wang, Jing Qiu, Lei Hou, Xiaojuan Zheng, Suihuai Yu

**Affiliations:** ^1^Northwestern Polytechnical University, China; ^2^University of Electronic Science and Technology of China, China

## Abstract

**Background:**

The analysis and comprehension of the coordination control of a human gait on common grounds benefit the development of robotic exoskeleton for motor recovery.

**Objective:**

This study investigated whether the common grounds effect the interjoint coordination of healthy participants with/without exoskeletons in walking.

**Methods:**

The knee-ankle coordination and hip-knee coordination of 8 healthy participants in a sagittal plane were measured on five kinds of pavements (tiled, carpet, wooden, concrete, and pebbled) with/without exoskeletons, using the continuous relative phase (CRP). The root mean square of CRP (CRP_RMS_) over each phase of the gait cycle is used to analyze the magnitude of dephasing between joints, and the standard deviation of CRP (CRP_SD_) in the full gait cycle is used to assess the variability of coordination patterns between joints.

**Results:**

The CRP_Hip-Knee/RMS_ of the carpet pavement with exoskeleton is different from that of other pavements (except the tiled pavement) in the midstance phase. The CRP_Hip-Knee/RMS_ on the pebble pavement without exoskeleton is less than that on the other pavements in all phases. The CRP_Hip-Knee/SD_ of the pebble pavement without exoskeleton is smaller than that of other pavements. The CRP_Knee-Ankle/SD_ with/without exoskeleton is similar across all pavements.

**Conclusion:**

The compressive capacity of the pavement and the unevenness of the pavement are important factors that influence interjoint coordination, which can be used as key control elements of gait to adapt different pavements for robotic exoskeleton.

**Novelty:**

We provide a basis of parameter change of kinematics on different common grounds for the design and optimization of robotic exoskeleton for motor recovery.

## 1. Introduction

The robotic exoskeleton provides assistance in time and replicates human walking at some extent. The interjoint coordination patterns of human walking are applied to the gait control for robotic exoskeleton. However, the gait of robotic exoskeleton for rehabilitation is usually fixed, and the robotic exoskeleton for rehabilitation cannot perceive ground changes. Although much is known about the intersegmental coordination of walking on the treadmill or uneven ground [1], the effect of common grounds such as the tiled ground on interjoint coordination has not been studied systematically.

The information of walking patterns, such as the coordination pattern between joints, provides basic data to classification and algorithm of gait control [2] for robotic exoskeleton. The robotic exoskeleton reduces the muscular effort compared to free walking [3, 4]. To increase walking efficiency of humans, it needs to reduce impact on the natural walking gait by minimizing changes in kinematics [5]. In addition, the appropriate assistive strategies constitute the human-robot motion, which benefits the assistive isotropy of the motion, and improves the assistive efficiency of the force [6]. Matching the assistance pattern of exoskeleton with the individual also needs to maximize the advantage of the device and minimize the human energy cost during walking [7].

The interjoint coordination in a sagittal plane was analyzed by the continuous relative phase (CRP) [8], which correlated temporal-spatial parameters [9] in joints and was used to evaluate the intersegment coordination [1, 10–12] as well as the interjoint coordination [8, 13, 14]. Human walking on different kinds of grounds seems to adopt different walking patterns through adjusting the joint kinematic. Still, the coordination patterns of a human body with exoskeletons normally imitate the coordination patterns of the human body without exoskeletons. The more similar the interjoint coordination patterns of robotic exoskeleton is to that of a normal person, the better for hemiplegic patients on motor recovery. It will be detrimental to the rehabilitation of hemiplegic patients if the tendency of the joint angle of the human body with/without exoskeleton is so different. Robotic lower limb exoskeletons have significant potential for gait assistance and rehabilitation [15]. However, we partly understand how people walking with robotic devices adapt to the daily living environment. Studying how an individual adapts or responds to different grounds in walking remains an open challenge [16, 17].

What is more, it is hard to find studies focusing on the effect of common grounds on joint kinematics when humans walk on different kinds of grounds with exoskeleton in daily life. Hence, in the current study, five kinds of pavements (tiled pavement, carpet pavement, wooden pavement, concrete pavement, and pebble pavement) were paved with real material in the experimental environment to figure out which joint the humans would adjust to adapt different pavements and to see if they adjust the patterns of joint kinematics to adapt different kinds of grounds. Based on CRP, the consistent proximal-to-distal coordination, such as hip-knee coordination and knee-ankle coordination, was measured with/without exoskeleton on five kinds of pavements across eight healthy participants in this study. We also expect the study of consistent proximal-to-distal coordination to provide support for the motion planning of robotic exoskeleton during walking on different kinds of grounds. The hypotheses of this study are as follows:

*Hypothesis 1:* when walking with exoskeletons on the five kinds of pavements, the pattern and variability of interjoint coordination would be similar between different pavements

*Hypothesis 2:* when walking without exoskeletons on the five kinds of pavements, there would be a significant difference between different pavements in the pattern and variability of interjoint coordination

## 2. Methods

Eight young and healthy participants (age: 23 ± 1.6 years, sex: male, leg length: 0.89 ± 0.03 m, mass: 76.6 ± 6.4 kg, and height: 172.6 ± 6.5 cm) were recruited to take part in the experiment with written informed consent before the experiment. All procedures were approved by the Sichuan Provincial Rehabilitation Hospital Review Board.

**Figure 1:**
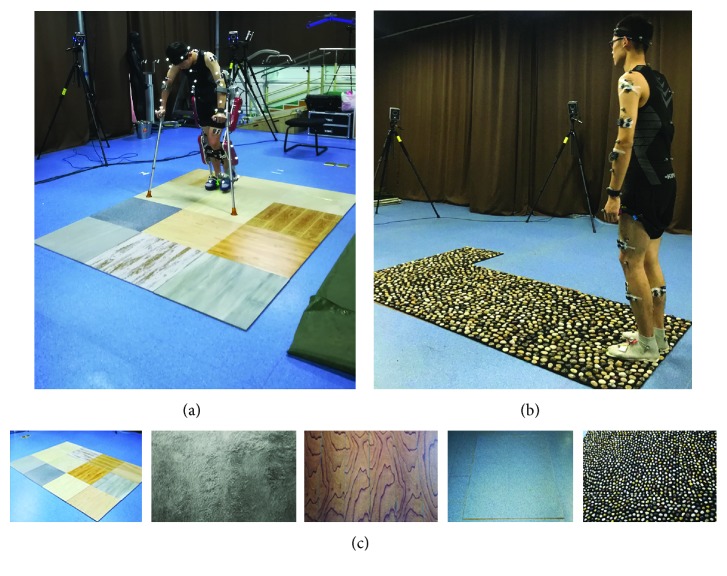
Experimental environment: (a) participant with exoskeleton walking on tiled pavement, (b) participant without exoskeleton walking on pebbled pavement, and (c) tiled pavement, carpet pavement, wooden pavement, concrete pavement, and pebbled pavement.

The kinematics data were captured by the VICON System (V5, Oxford, VICON, UK) with 8 infrared cameras at 100 Hz. The human-exoskeleton system marker set (Figure 1(a)) was a modification of a marker set in the VICON system. The human and exoskeleton were regarded as a whole system in the modification of the marker set, so markers placed on the human's pelvis, legs, ankles, and heels are moved to the exoskeleton's pelvis, legs, ankles, and heels. Thirty-nine reflective markers were placed on the human-exoskeleton system, including the seventh cervical vertebrae, sternum, shoulders, elbows, anterior-superior iliac spine, exoskeleton thighs, exoskeleton knees, exoskeleton shanks, exoskeleton ankles, 2^nd^ metatarsal heads, and exoskeleton heels. In addition, four markers were stuck on the headband and two markers were stuck on the wristband.

The lower limb exoskeleton called AIDER (Figure 1(a)) is developed by our lab, which can assist walking for T7-T12 SCI patients with a height of 160-185 cm. The main controller and battery are set on the back. Two motors are, respectively, fixed on the unilateral hip joint and the knee joint to provide active drives, and one spring is fixed on the ankle joint to provide passive drives. Two adjustable crutches with two keys interacting with the main controller wirelessly assist the balance of the human-exoskeleton system. The interfaces between AIDER and the participant's body are two foot bindings, two bands tied to the front protection pad to constraint the calf, two bands tied to the back protection pad to constraint the thigh, and two buckled waist belts limiting the upper body in it. AIDER (8 degrees of freedom, 26 kg) allows patients to walk at the speed of 0.03 m/s-0.9 m/s.

Five typical pavements (Figure 1(c)) are made of real materials. The sizes of all simulated surfaces with different friction coefficients (Table 1) are 3 m by 1 m. Pavements were tiled pavement, carpet pavement, wooden pavement, concrete pavement, and pebble pavement. Participants first walked without exoskeleton on the ranked pavements for 2 meters for 4 times at normal speed, and then, they walked with exoskeleton on the pavements at normal speed for 2 meters for 4 times after at least 1-hour training. To ensure the safety of participants, a researcher followed the participants' walking with exoskeleton throughout the whole experiment.

The gait cycle from heel strike to heel strike was determined by the trajectory of heel markers. All variables were normalized from 0 to 1, compared with a stride cycle. Each joint's angle in a sagittal plane was interpolated to the same quantity in one gait cycle. The angular velocity of each joint was derived from the differentiation of angle displacement. The phase angle is equal to the arctangent of the ratio of the normalized angular velocity to the normalized angular displacement, and CRP is equal to the phase angle of the proximal joint minus the phase angle of the distal joint [9, 11, 14]. The root mean square of CRP (CRP_RMS_) was selected to analyze the magnitude of dephasing between joints at a specific phase of the gait cycle, and the standard deviation of CRP (CRP_SD_) was selected to assess the variability of the coordination pattern between joints in the full gait cycle [9]. Peak ankle dorsiflexion in the midstance, peak ankle plantar flexion in the late stance, peak ankle dorsiflexion in swing, peak knee flexion in swing, peak hip extension in the late stance, and peak hip flexion in swing were selected as six key parameters for the kinematic analysis. All data were processed by MATLAB (MathWorks, Natick, MA, USA). To examine the changes in kinematics across one gait cycle for ankle, knee, and hip joints, the paired *t*-test was used to analyze the statistical significance of gait parameters between pavements by SPSS (v25, IBM Corp., Armonk, USA). The value of significance level was set at an alpha value of 0.05.

## 3. Results

### 3.1. Joint Kinematics

In a gait cycle, the trends of hip, knee, and ankle angles of the human system are not exactly the same as normal people. The overall angle of the hip, knee, and ankle joints of the human-machine system is much smaller than that of a normal person. Peak ankle dorsiflexion with exoskeleton in the midstance phase is larger than that without exoskeleton on five kinds of pavements (Table 2). With exoskeleton, there is a significant difference in the peak ankle dorsiflexion in the midstance between the carpet pavement and the pebble pavement (paired *t*-tests, *p* = 0.009). Without exoskeleton, the peak ankle plantar flexion (paired *t*-tests, *p* = 0.031) in the late stance phase has a significant difference between the pebble pavement and the carpet pavement. Similarly, without exoskeleton, the peak ankle plantar flexion (paired *t*-tests, *p* = 0.043) in the late stance phase has a significant difference between the pebble pavement and the wooden pavement. The ungiven results of paired *t*-test of peak values with/without exoskeleton between pavements indicate no significant difference.

**Figure 2:**
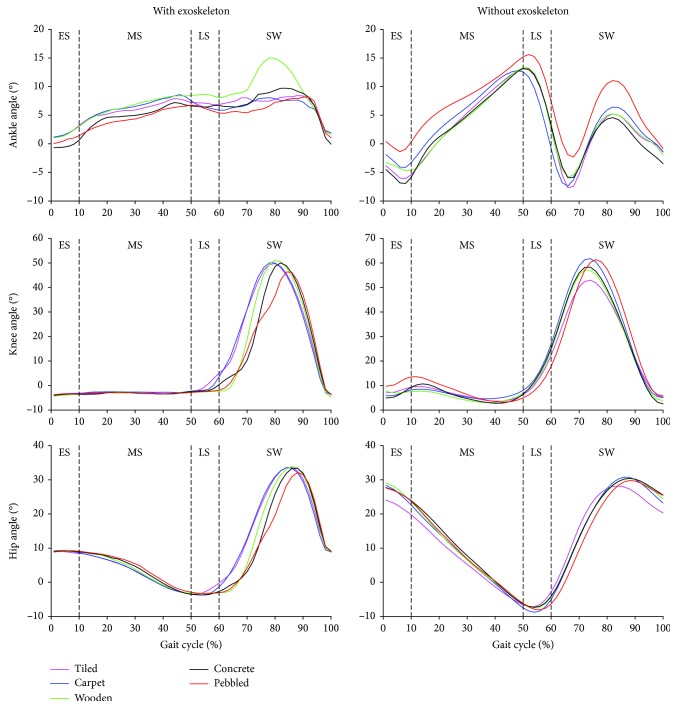
Changes in kinematics at the ankle, knee, and hip. Mean angle of the ankle, knee, and hip in a sagittal plane for participants (*n* = 8) with/without exoskeleton over the gait cycle on each kind of pavements. The gait cycle is from the heel strike to the next heel strike of the left foot. ES = early stance; MS = midstance; LS = late stance; SW = swing phase.

On five types of pavements, the trends (see Figure 2) of the joint angle of the human-exoskeleton system are significantly different from the trends of the joint angle without exoskeleton. The ankle angle with exoskeleton over the gait cycle (except the early stance phase) on the pebble pavement is the smallest among the five kinds of pavements, but the ankle angle without exoskeleton over the gait cycle on the pebble pavement is the largest among the five kinds of pavements. With/without exoskeleton, the knee angle in the stance phase tends to be consistent on the five kinds of pavements. On the contrary, the knee angle in the stance phase with/without exoskeleton tends to be different in the five kinds of pavements. Although the hip angle with exoskeleton in the stance phase on the pebble pavement is almost larger than that on the other pavements, the hip angle with exoskeleton in the first half of the swing phase on the pebble pavement is smaller than the hip angle with exoskeleton on the other pavements. This trend is similar to the hip angle without exoskeleton.

### 3.2. Measurement of Interjoint Coordination

**Figure 3:**
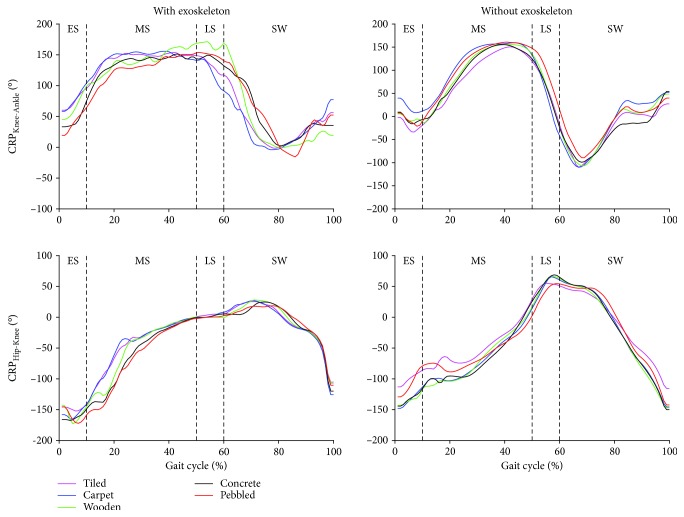
Continuous relative phase (CRP) patterns between the knee and ankle and between the hip and knee in the sagittal plane. Mean CRP for participants (*n* = 8) with/without exoskeleton over the gait cycle on each kind of pavements. The gait cycle is from the heel strike to the next heel strike of the left foot. ES = early stance; MS = midstance; LS = late stance; SW = swing phase.

This study explored the effects of different pavements on coordination patterns, using the root mean square of CRP. RMS values indicate the magnitude of the dephasing between two adjacent joints but not on which joint precedes [12]. However, the CRP curves (Figure 3) provide which joint precedes on the specific pavement with/without exoskeleton: the knee precedes the ankle at all phases of the gait cycle on pavements (except the pebble pavement in the swing phase) with exoskeleton, and the hip precedes the knee in the stance phase on all pavements with exoskeleton. The knee precedes the ankle in the midstance phases on pavements without exoskeleton, and the ankle precedes the knee in the early stance phase on pavements (except the carpet pavement) without exoskeleton. The knee precedes the hip in the early stance phase and in the midstance phase on all pavements without exoskeleton, while the hip precedes the knee in the late stance phase on all pavements without exoskeleton.

The CRP_Hip-Knee/RMS_ on the pebble pavement with exoskeleton is larger than that on the other pavements in the early stance phase and in the midstance phase. On the contrary, the CRP_Hip-Knee/RMS_ on pebbled pavement without exoskeleton is less than that on the other pavements in all phases, while the CRP_Hip-Knee/RMS_ on the tiled pavement without exoskeleton is less than that on the other pavements in all phases (as seen in Table 3). With exoskeleton, the CRP_Hip-Knee/RMS_ in the midstance phase has a significant difference between the carpet pavement and the wooden pavement (paired *t*-tests, *p* = 0.034), between the carpet pavement and the concrete pavement (paired *t*-tests, *p* = 0.028), and between the carpet pavement and the pebble pavement (paired *t*-tests, *p* = 0.044). Moreover, the CRP_Hip-Knee/RMS_ with exoskeleton in the late stance phase has a significant difference between the wooden pavement and the pebble pavement (paired *t*-tests, *p* = 0.029) and in the swing phase between the carpet pavement and the wooden pavement (paired *t*-tests, *p* = 0.024). Without exoskeleton, the CRP_Hip-Knee/RMS_ in the early stance phase has a significant difference between the tiled pavement and the concrete pavement (paired *t*-tests, *p* = 0.02) and between the wooden pavement and the pebble pavement (paired *t*-tests, *p* = 0.009). In addition, the CRP_Hip-Knee/RMS_ without exoskeleton in the late stance phase has a significant difference between the carpet pavement and the pebble pavement (paired *t*-tests, *p* = 0.033) and between the concrete pavement and the pebble pavement (paired *t*-tests, *p* = 0.033).

The CRP_Knee-Ankle/RMS_ on the tiled pavement with exoskeleton is larger than that on the other pavements in the early stance phase and in the midstance phase, while the CRP_Knee-Ankle/RMS_ on the tiled pavement with exoskeleton is the less than that on the other pavements in the late stance phase and in the swing phase. On the contrary, the CRP_Knee-Ankle/RMS_ on the pebble pavement with exoskeleton is the less than that on the other pavements in the early stance phase and in the midstance phase, while the CRP_Knee-Ankle/RMS_ on the tiled pavement with exoskeleton is larger than that on the other pavements in the late stance phase and in the swing phase. The CRP_Knee-Ankle/RMS_ on the pebble pavement without exoskeleton is less than that on the other pavements in the early stance phase and in the swing phase, while the CRP_Knee-Ankle/RMS_ on the tiled pavement without exoskeleton is larger than that on the other pavements in the midstance phase and late stance phase. The CRP_Knee-Ankle/RMS_ on the carpet pavement without exoskeleton is larger than that on the other pavements in the early stance phase and in the midstance phase (as seen in Table 3). With exoskeleton, there is a significant difference of the CRP_Knee-Ankle/RMS_ between the carpet pavement and the pebble pavement in the late stance phase (paired *t*-tests, *p* = 0.027) and in the swing phase (paired *t*-tests, *p* = 0.026). Without exoskeleton, there is a significant difference of the CRP_Knee-Ankle/RMS_ in the midstance phase between the tiled pavement and the carpet pavement (paired *t*-tests, *p* = 0.01) and in the late stance phase between the concrete pavement and the pebble pavement (paired *t*-tests, *p* = 0.048).

With exoskeleton, there is a significant difference of the CRP_Hip-Knee/SD_ between the carpet pavement and the wooden pavement (paired *t*-tests, *p* = 0.024) in the full gait cycle. Without exoskeleton, there is a significant difference of the CRP_Hip-Knee/SD_ in the full gait cycle between the tiled pavement and the concrete pavement (paired *t*-tests, *p* = 0.029), between the tiled pavement and the pebble pavement (paired *t*-tests, *p* = 0.033), between the carpet pavement and the pebble pavement (paired *t*-tests, *p* = 0.015), and between the wooden pavement and the pebble pavement (paired *t*-tests, *p* = 0.005). The trends of CRP with exoskeleton oscillate more frequently than the trends of CRP without exoskeleton over the gait cycle on the pavements.

## 4. Discussion

Our results suggest that the common pavements cause a significant difference of interjoint coordination with/without exoskeleton only in some phases of the gait cycle, so the hypothesis 1 and the hypothesis 2 are only partially proved. The compressive capacity of the carpet pavement is obviously lower than the other pavements, which may cause the difference of CRP_Hip-Knee/RMS_ with exoskeleton between the carpet pavement and other pavements (except the tiled pavement) in the midstance. Moreover, the compressive capacity of the carpet pavement may cause the difference of CRP_Knee-Ankle/RMS_ with exoskeleton between the carpet pavement and the pebble pavement in the late stance phase and in the swing phase. However, the unevenness of pebble pavement as another influencing factor should not be ignored. Because the unevenness of the pebble pavement increases the physical energy consumption [18], the CRP_Hip-Knee/RMS_ of the pebble pavement without exoskeleton is lower than the other pavements and statistically different from the carpet pavement and the concrete pavement. The unevenness of the pebble pavement may induce the cautious dynamic neuromuscular control [13] of participants and enhance their leg stiffness [1, 10] so that the CRP_Hip-Knee/SD_ of the pebble pavement without exoskeleton is smaller than that of other pavements and statistically different from that of other pavements (except concrete pavement). When walking on pavements with exoskeleton, participants need to adjust the center of gravity to keep the human-exoskeleton system balance with the help of crutches and prepare for the next step in stance, which may cause the difference of interjoint coordination patterns with exoskeleton between pavements.

The exoskeleton was set in a fixed gait and joint moment, so the peak values should be similar between pavements. However, the peak ankle dorsiflexion of walking on the carpet pavement in the midstance is significantly different from that of walking on the pebble pavement, which may due to the active intervention from participants on the ankle. When the participants without exoskeleton walk on the pavements, it is only found that the peak ankle plantar flexion in the late stance phase on the pebble pavement is significantly different from that on the carpet pavement and the wooden pavement. This result indicates that the friction coefficients of pavements do not impose on gait parameters in kinematics, but the unevenness of pavements obviously affects the gait parameters in kinematics [1, 10]. From the peak values without exoskeleton at all pavements, the human mainly adjusts the ankle dorsiflexion in the swing phase to adapt common pavements. Due to the fixed gait and joint moment of exoskeleton, the conditions that the knee precedes the ankle without exoskeleton on all pavements in the early stance and in the swing phase were reversed. Similarly, the conditions that the hip precedes the knee without exoskeleton on all pavements in the late stance phase were also reversed (Figure 3).

## 5. Conclusions and Limitations

In summary, our work reveals the effect of common pavements on interjoint coordination with/without exoskeleton. The compressive capacity of the pavement and the unevenness of pavement are important factors that influence the interjoint coordination. The compressive capacity of the pavement can modify the magnitude of dephasing between the hip and knee with exoskeleton in the midstance phase and in the swing phase. The unevenness of the pavement can change the magnitude of dephasing between the hip and the knee without exoskeleton in the early stance phase and in the late stance phase and increases the stability of the coordination pattern between the hip and the knee without exoskeleton. The finding suggests that the identification of the compressive capacity and the unevenness of common grounds should be used for the control strategy of exoskeleton to enhance the coordination and benefit the motor rehabilitation.

There are three limitations that need to be considered. First, the effects of physiological characteristics such as age, gender, and weight on the gait parameters of different pavement kinematics have not been included. Second, there are not only random displacements between the human body and the exoskeleton but also individual differences between human bodies, which make it difficult for the human-exoskeleton model to measure the exact gait parameters of human-exoskeleton. Therefore, in exoskeleton experiments, there are uncertain errors in the gait parameters of human-exoskeleton. Third, this study did not explore muscle adaptation and joint kinetics of people who may adapt to different friction coefficients. Future work will focus on the effects of different friction coefficient pavements on muscle adaptation and joint dynamics, which can further explain how people adapt to pavements with different coefficients of friction.

## Figures and Tables

**Table 1:** Friction coefficients of pavements.

Pavements	Tiled	Carpet	Wooden	Concrete	Pebbled
Coefficient of frictions	0.32	0.15	0.33	0.34	0.20

**Table 2:** Gait parameters with/without exoskeleton at five kinds of pavements.

	With exoskeleton					Without exoskeleton				
	Tiled	Carpet	Wooden	Concrete	Pebbled	Tiled	Carpet	Wooden	Concrete	Pebbled
Peak ankle dorsiflexion in midstance (°)	9 ± 5	9 ± 4	6 ± 5	6 ± 6	8 ± 4	14 ± 3	13 ± 5	14 ± 4	14 ± 2	12 ± 8
Peak ankle plantar flexion in late stance (°)	N	N	N	N	N	9 ± 8	10 ± 7	9 ± 7	7 ± 8	3 ± 7
Peak ankle dorsiflexion in swing (°)	10 ± 5	10 ± 4	10 ± 12	8 ± 6	9 ± 5	6 ± 5	7 ± 6	6 ± 5	6 ± 6	9 ± 8
Peak knee flexion in swing (°)	34 ± 1	34 ± 1	21 ± 18	26 ± 16	34 ± 1	34 ± 11	31 ± 9	31 ± 8	31 ± 8	23 ± 16
Peak hip extension in late stance (°)	4 ± 2	4 ± 2	2 ± 2	3 ± 3	3 ± 2	9 ± 6	10 ± 7	8 ± 6	8 ± 7	6 ± 7
Peak hip flexion in swing (°)	34 ± 1	34 ± 1	21 ± 18	26 ± 16	34 ± 1	34 ± 11	31 ± 9	31 ± 8	31 ± 8	23 ± 16

Peak values as the mean ± standard deviation; N: no data.

**Table 3:** Coordination: CRP root mean square (CRP_RMS_) and variability (CRP_SD_) over the full gait cycle for participants (*n* = 8) with/without exoskeleton over the gait cycle on each kind of pavements.

	With exoskeleton					Without exoskeleton				
	Tiled	Carpet	Wooden	Concrete	Pebbled	Tiled	Carpet	Wooden	Concrete	Pebbled
	CRP_Hip-Knee/RMS_									
Early stance	148 ± 33	155 ± 18	158 ± 22	161 ± 27	162 ± 15	139 ± 20	313 ± 20	133 ± 30	130 ± 21	107 ± 37
Midstance	55 ± 27	53 ± 20	67 ± 30	77 ± 31	83 ± 31	82 ± 25	79 ± 22	79 ± 17	80 ± 21	52 ± 35
Late stance	6 ± 8	5 ± 5	1 ± 1	4 ± 6	2 ± 1	62 ± 21	56 ± 14	56 ± 10	58 ± 20	44 ± 20
Swing	34 ± 7	38 ± 6	34 ± 7	35 ± 8	33 ± 7	74 ± 5	71 ± 9	73 ± 11	72 ± 8	71 ± 9

	CRP_Knee-Ankle/RMS_									
Early stance	82 ± 63	86 ± 59	77 ± 61	56 ± 58	50 ± 47	38 ± 47	42 ± 26	40 ± 33	28 ± 13	29 ± 14
Midstance	148 ± 50	149 ± 47	147 ± 39	141 ± 59	136 ± 57	120 ± 14	133 ± 13	125 ± 17	123 ± 15	130 ± 11
Late stance	138 ± 60	129 ± 52	166 ± 13	146 ± 50	151 ± 58	87 ± 41	90 ± 20	93 ± 25	86 ± 27	112 ± 32
Swing	58 ± 30	50 ± 24	70 ± 11	71 ± 26	82 ± 28	75 ± 11	74 ± 12	74 ± 13	72 ± 28	59 ± 20
CRP_Hip-Knee/SD_	55 ± 13	57 ± 9	60 ± 11	63 ± 11	65 ± 10	75 ± 6	71 ± 7	71 ± 8	71 ± 9	63 ± 9
CRP_Knee-Ankle/SD_	64 ± 16	64 ± 16	71 ± 5	63 ± 21	67 ± 13	89 ± 8	90 ± 8	89 ± 9	87 ± 15	84 ± 11

Root mean square (RMS) as the mean ± standard deviation. (0–10%) data points in one gait cycle for each participant, (10–50%) data points in one gait cycle for midstance, (50–60%) data points in one gait cycle for late stance, and (60–100%) data points in one gait cycle for the swing phase.

## Data Availability

The data that support the findings of this study are available on request from the corresponding author, Jing Qiu.
